# Potential diagnostic challenges of intracerebral hemorrhage as an index presentation of metastatic choriocarcinoma: A case series

**DOI:** 10.1002/ccr3.8835

**Published:** 2024-04-26

**Authors:** Seth Kyei‐Fram, Osei Yaw Asamoah, Martin Agyei, Priscilla Abrafi Opare‐Addo

**Affiliations:** ^1^ Komfo Anokye Teaching Hospital Kumasi Ghana; ^2^ Kwame Nkrumah University of Science & Technology Kumasi Ghana

**Keywords:** brain metastasis, case series, gestational trophoblastic neoplasia, hemorrhagic stroke

## Abstract

**Key Clinical Message:**

In young women presenting with atypical features of intracerebral hemorrhage, metastatic choriocarcinoma should be considered as a differential diagnosis. In resource‐poor settings, a high index of suspicion and serum β‐hCG are crucial for diagnosis.

**Abstract:**

Intracerebral hemorrhage in the young is rarely caused by metastatic choriocarcinoma. Diagnosis of this condition may be particularly challenging in resource‐poor settings where access to diagnostic technologies may be limited. We present a case series of three young females diagnosed with metastatic choriocarcinoma after initially presenting with intracerebral hemorrhage, each demonstrating unique clinical manifestations. We aim to highlight the diagnostic considerations in the management of this infrequently encountered cause of intracerebral hemorrhage, especially in resource‐constrained settings. Case 1 involved a 21‐year‐old woman who was initially diagnosed with intracerebral hemorrhage likely of tumoral origin from an unknown primary source. Further evaluation revealed extremely high levels of β‐hCG and features suggestive of an intrauterine malignancy, which led to a diagnosis of metastatic choriocarcinoma. This further became complicated by pulmonary embolism. Unfortunately, she succumbed to respiratory failure during treatment. Case 2 is a young woman who presented to the emergency unit and was managed as a case of lobar intracerebral hemorrhage. Further checks revealed a previous history of hysterectomy done on account of placental site trophoblastic tumor, which promoted an evaluation for choriocarcinoma. Case 3 involved a 20‐year‐old patient who initially presented with headache and vomiting. An enhanced magnetic resonance imaging showed a large subacute right temporal occipital subependymal hemorrhage with mass effect. After probing further, we discovered that she underwent exploratory laparotomy for suspected ruptured ectopic gestation, which later turned out to be a gestational trophoblastic neoplasia. After further evaluation a diagnosis of choriocarcinoma with brain metastasis. Our case series emphasizes the importance of having a high index suspicion in young females who present with atypical features of ICH. The varied clinical scenarios highlight the challenges in diagnosing young females. It also underscores the critical role of serum β‐hCG, especially in resource‐limited settings where biopsies are not readily available. Building a repository of these diverse manifestations is essential for increasing the index of suspicion and ultimately improving patient outcomes.

## 1 INTRODUCTION

Choriocarcinoma is a rare and highly aggressive malignancy that develops from trophoblastic cells. There are two major forms of choriocarcinoma – gestational and non‐gestational choriocarcinoma.

Non‐gestational choriocarcinoma originates from germ cells outside of the context of pregnancy. It can arise in the gonads (ovaries or testes) or extragonadal sites (such as the mediastinum, retroperitoneum, or pineal gland).^1^

On the other hand, gestational choriocarcinoma arises from abnormal trophoblastic tissue during pregnancy. It most commonly occurs following a molar pregnancy (complete or partial hydatidiform mole) or a normal pregnancy. Gestational choriocarcinoma occurs in approximately 1 in 20,000–40,000 pregnancies. About half of gestational choriocarcinomas occur after molar pregnancies; the remainder occur following a spontaneous abortion, ectopic pregnancy, or a term pregnancy.^2^ Approximately 30% of cases of choriocarcinoma are diagnosed with metastatic disease. The lungs (80%) are the most common site of metastasis, followed by the vagina (30%) and the liver (10%).^3^

In a study conducted by Savage et al., findings indicated that non‐molar choriocarcinoma exhibited an increased tendency for cerebral metastasis compared to molar choriocarcinoma. Specifically, they noted central nervous system (CNS) involvement in 1 out of every 22,000 instances of molar pregnancies, whereas approximately 20% of non‐molar choriocarcinoma cases presented CNS involvement. Consequently, the overall risk of developing gestational trophoblastic neoplasia (GTN) with brain metastases was estimated to be approximately 2–3 cases per million pregnancies. Even though choriocarcinoma is highly aggressive, it is very responsive to chemotherapy.^2^, ^4^ Therefore, the importance of early detection cannot be overemphasized.^5^

In young people, structural lesions such as arteriovenous malformations and aneurysms are typically the cause of lobar intracerebral hemorrhage (ICH), whereas hypertension is typically the cause of non‐lobar ICH.^6^ We present three cases of choriocarcinoma with brain metastases who presented with intracranial hemorrhage. The case series is to draw attention to metastatic choriocarcinoma as a cause of unexplained ICH in women of childbearing age.

## 2 CASE 1

### 2.1 Case history and examination

A 21‐year‐old woman, Para 1(A) + 1 SA presented with headache and blurred vision of 3 weeks duration. The patient had no known history of any chronic medical condition. The headache was global, of gradual onset, throbbing in character, and relieved with analgesics.

She was oriented and had nuchal rigidity with anisocoria; the left pupil was 4 mm, unreactive to light, and the right was 2 mm reactive to light; there was ptosis of the left eye with subconjunctival hemorrhage. She had a normal tone and full power in all limbs.

On admission day, two patient was noticed to be bleeding per vagina with a mass protruding from the vagina. On further inquiry, the patient had experienced a spontaneous abortion 2 months before presentation, for which evacuation of the uterus was done three times at a peripheral facility.

### 2.2 Differential diagnosis, investigations, and treatment

**FIGURE 1:**
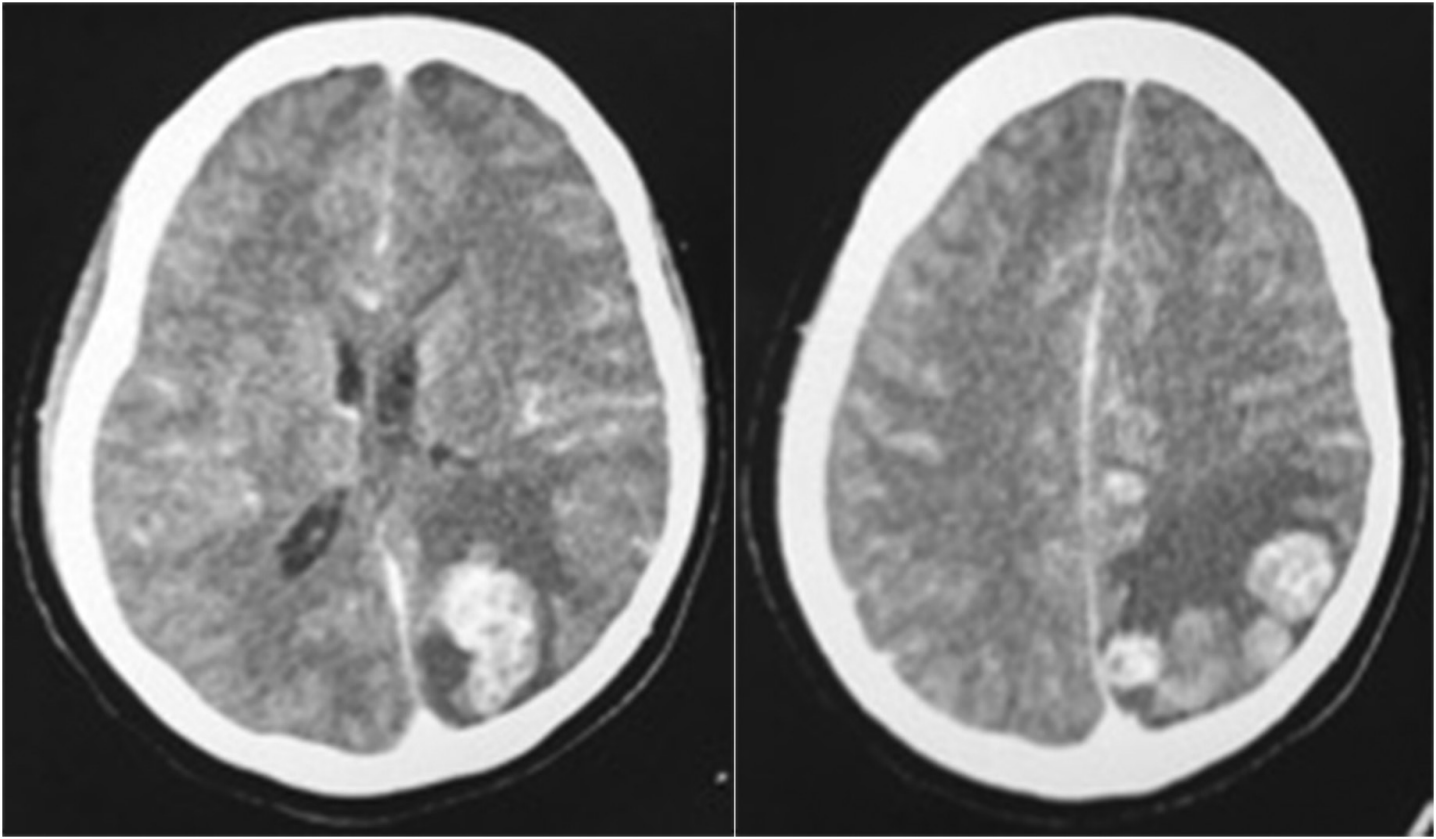
Case 1. Contrast‐enhanced head CT showed heterogeneously enhancing slightly hyperdense nodular masses of varying sizes with moderate perilesional edema in the left occipitoparietal region.

A contrast‐enhanced brain CT showed an enhancing left ocular and left occipital hyperdensity with associated mass effect (Figure 1). A diagnosis of left ocular and left occipital hypervascular metastases from an unknown primary was made.

The uterus measured 8.3 × 7.2 × 6.4 cm on abdominal ultrasonography, with a heterogenous endometrial collection measuring 6.4 × 4.4 × 2.7 cm (volume – 40.4 mLs) characterized by echogenic and anechoic areas. The collection showed internal vascularity on color Doppler ultrasound. A urine pregnancy test was positive. Quantitative serum β‐hCG was greater than 15,000 mIU/mL (normal value: 5–25 mIU/mL). A diagnosis of FIGO stage IV choriocarcinoma with possible intracerebral metastases was established. The patient was transferred to the gynecological‐oncology ward to be evaluated by the oncology unit and further management.

### 2.3 Outcome and follow‐up

Unfortunately, the patient developed left hemiparesis with a decline in consciousness on day six of admission. On day seven of admission, the patient had an acute episode of respiratory distress. On evaluation, a diagnosis of acute pulmonary embolism was made. The patient was started therapeutic heparin dose. However, the patient succumbed to respiratory failure. The family did not consent to an autopsy.

## 3 CASE 2

### 3.1 Case history and examination

The patient is a 31‐year‐old woman, Para 2 (A) + 0, who presented with dizziness and a transient right hemiparesis of a day's duration. She was oriented and had no residual weakness at the time of evaluation; her pupils were round and of equal size, with sensitivity to light. The patient was admitted to the neurology ward.

Upon further review of the gynecological history, it was noted that the patient had a spontaneous abortion 3 months prior to the presentation and had a hysterectomy done on account of a placental site trophoblastic tumor (PSTT). Serum human placental lactogen level was requested, however, it was not carried out because the patient could not afford it, which presents a limitation.

### 3.2 Differential diagnosis, investigations, and treatment

Contrast‐enhanced brain CT showed an irregular, non‐enhancing hyperdense lesion with moderate perilesional hypodensity consistent with acute hemorrhage measuring about 3.4 × 1.6 cm seen in the left parietal region. This prompted the team to evaluate for metastatic GTN. The result of the quantitative serum β‐hCG requested was greater than 15,000 mIU/mL (normal value: 5–25 mIU/mL). Abdominopelvic ultrasound was unremarkable, chest x‐ray showed features suggestive of pulmonary metastases with right pleural effusion, volume of 298 mLs on ultrasound. A diagnosis of FIGO stage IV choriocarcinoma with possible intracerebral metastases was established, and she was admitted to the gynecological oncology ward.

The patient underwent assessment by the oncology team and commenced chemotherapy treatment comprising cisplatin and etoposide. She had a total of 4 cycles of chemotherapy, with the first 2 cycles administered at 25% dose reduction for both agents, followed by a dose of 100 mg/m^2^ for both agents for the subsequent 2 cycles.

### 3.3 Outcome and follow‐up

She was discharged to home to continue with chemotherapy. Serum β‐hCG after 4 months of chemotherapy was 3.829 mIU/mL. The patient has not experienced any complications from the therapy.

## 4 CASE 3

### 4.1 Case history and examination

A 20‐year‐old reported to the hospital with a week's duration of headache, which was associated with vomiting. The headaches were more intense in the temporal regions and were burning in character. The patient presented to the hospital with a 24‐h history of confusion. On examination, she was aggressive and required sedation.

Further inquiries revealed a rather interesting gynecological history. Three months before presenting, she had generalized abdominal pain, was diagnosed with peritonitis, and was scheduled for a laparotomy. A urine pregnancy test performed as she was being prepared for surgery was positive. She experienced an episode of bleeding per vagina, which caused the team to suspect a ruptured ectopic gestation and peritonitis. An exploratory laparotomy was performed; the notable finding during the procedure was a peri‐renal hematoma. A suspicion of GTN was made when a serum β‐hCG requested post‐laparotomy was 11,293 mIU/mL. In addition, an abdominopelvic ultrasound showed a right renal subcapsular hematoma with free‐intraabdominal fluid and minimal bilateral effusion and no sonographic evidence of pregnancy, endometrial mass, or ectopic gestation seen. She was discharged on postoperative day 5 with a referral to gynecology on an outpatient basis.

However, she was lost to follow‐up care and presented to the emergency department 3 months after the previous discharge with complaints of severe headaches.

### 4.2 Differential diagnosis, investigations, and treatment

**FIGURE 2:**
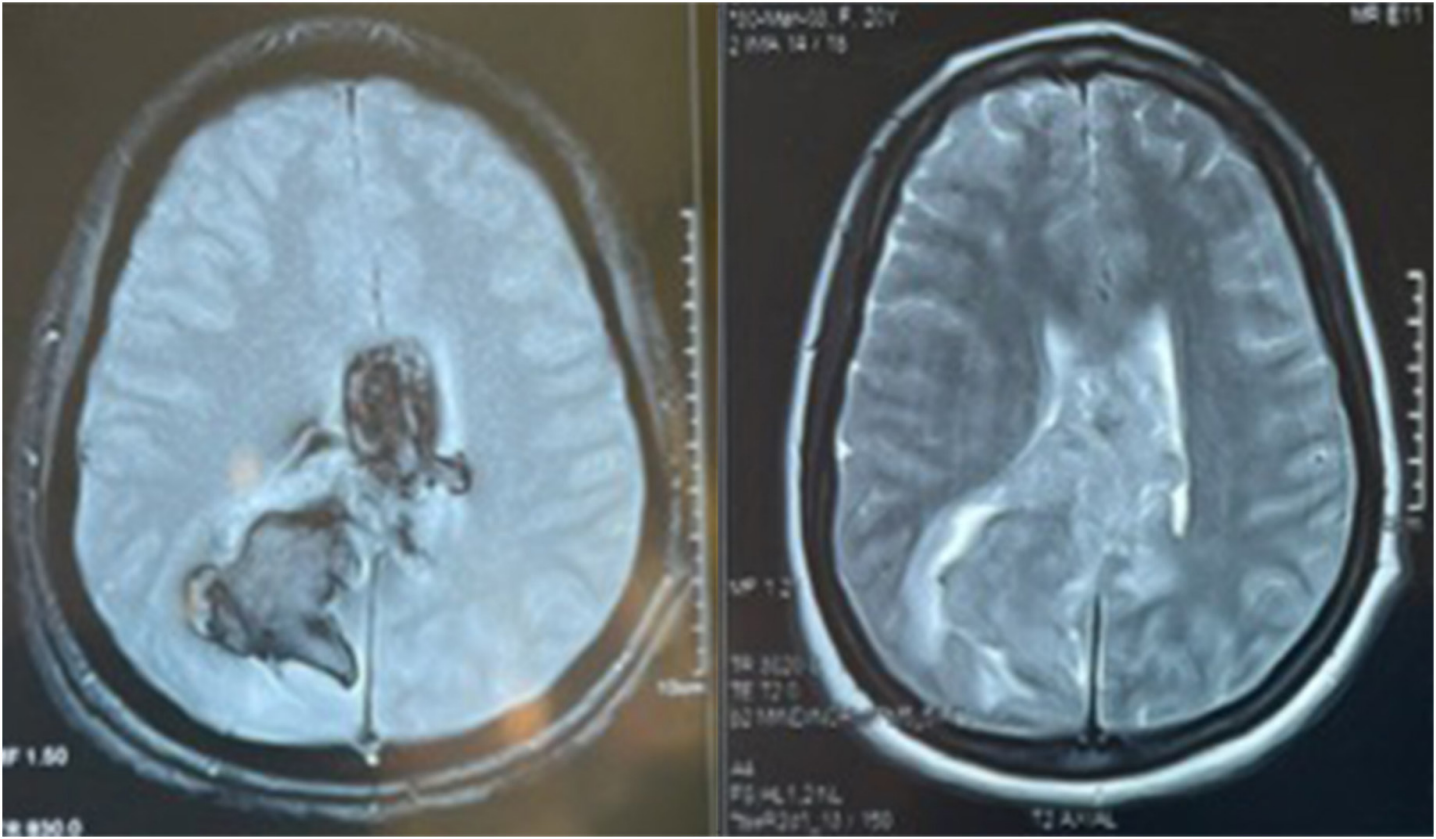
Case 3. MRI of the brain showed a large subacute right temporo‐occipital subependymal hemorrhage, which measures approximately 5.5 × 4.2 cm with intraventricular extension and exhibiting minimal perilesional edema.

An enhanced magnetic resonance imaging showed a large subacute right temporal occipital subependymal hemorrhage, which measures 5.5 × 4.2 cm with intraventricular extension and minimal mass effect (Figure 2). Due to her young age and the location of the lesion, arteriovenous malformation and aneurysmal rupture were suspected as the cause of the ICH. However, she required a magnetic resonance angiography study to exclude arteriovenous malformation as a cause. An abdominopelvic ultrasound showed bilateral renal parenchymal disease (Grade 3), normal pelvic ultrasound with no intrauterine or extrauterine gestation seen. Serum β‐hCG requested was 210,066 mIU/mL.

Based on the medical history, a diagnosis of FIGO stage IV choriocarcinoma with possible intracerebral metastases was made. The oncology unit scheduled her to undergo whole‐brain radiation therapy (WBRT) and chemotherapy comprising cisplatin and etoposide. She had 5 cycles of WBRT at a radiation dose of 20 Gy after which she started chemotherapy. She had 4 cycles of chemotherapy with cisplatin and etoposide at a dose of 100 mg/m^2^. She was also prescribed dexamethasone, omeprazole, and ibuprofen.

### 4.3 Outcome and follow‐up

She was discharged to home to continue with chemotherapy. The patient has no known complications from the therapy.

## 5 DISCUSSION

We presented three cases of metastatic choriocarcinoma manifesting as primary intracerebral hemorrhage (ICH). Choriocarcinoma is a highly malignant tumor that develops from trophoblastic cells. As observed in our cases, choriocarcinoma usually presents within 6 months following either a normal or an abnormal pregnancy. However, there seems to be no specific time interval between the preceding pregnancy and the diagnosis of GTN, which presents a diagnostic challenge. In their review, Mangla et al. reported that the interval between the initial pregnancy and the onset of choriocarcinoma ranged from 4 weeks to as far as 25 years.^3^

Though the patients had a history of pregnancy losses within the past 3 months, this vital information was not elucidated when they initially presented at the emergency. It is therefore imperative that in resource‐poor settings where imaging is not readily available, thorough history including gynecological history and examination be employed to aid in diagnosis. Routine urine pregnancy test should also be done for all women in their reproductive age, as well as serum β‐hCG when indicated.

Patients may present with abnormal uterine bleeding, abdominal pain, uterine subinvolution, or neurological deficits as has been described in the cases presented.^7^ Choriocarcinoma has a high propensity to spread to other organs with a predilection for the lung (94%), vagina (44%), liver (28%), and brain (28%).^8^

About 10% of choriocarcinoma cases would have brain metastasis, occurring in the parietal lobes, the temporal lobes, and the frontal lobes, in decreasing order.^9^ Patients with advanced disease typically exhibit cerebral involvement, and nearly all patients with brain metastasis also have concurrent pulmonary or vaginal involvement or both. Tumor emboli from the lungs are suspected to be the cause of brain metastases. The most prevalent way that cerebral involvement manifests itself is intracranial hemorrhage, which accounts for two‐thirds of all brain metastases. Brain involvement may lead to headache, nausea, vomiting, hemiparesis, altered level of consciousness, and seizures.^9^

Early detection and treatment of metastatic choriocarcinoma offers a good prognosis, with reports of complete resolution following chemotherapy.^4^, ^10^ However, clinical presentation and radiological features cannot distinguish choriocarcinoma and primary ICH. As was seen in the cases presented, all the patients were initially diagnosed with primary ICH and admitted to the neurology ward. It is therefore important that, in women of childbearing age who present with atypical intracerebral hemorrhage, metastatic choriocarcinoma be included in the differential diagnosis. The physician, therefore, should have a high index of suspicion.

Elevated levels of β‐hCG in a patient who has a history of either a normal or an abnormal pregnancy point to the diagnosis of choriocarcinoma.^2^, ^11^ As seen in our cases, the diagnosis was based on clinical signs and symptoms, radiological imaging, and high levels of serum β‐hCG. Serum β‐hCG is an important marker in the diagnosis of choriocarcinoma.

Choriocarcinoma has been found to exhibit a strong correlation with serum *β*‐hCG levels, with the degree of *β*‐hCG increase directly mirroring the tumor size and disease severity.^12^, ^13^ Choriocarcinoma originates from the cytotrophoblast and syncytiotrophoblast cells and as such exhibit significant β‐hCG production, with levels typically ranging from 100 to over 100,000 mIU/mL. In contrast, patients with PSTT exhibit low levels of *β*‐hCG; however, there is increased expression of hPL both on histologic slides and in serum.^14^, ^15^

Besides clinical and radiological imaging, the ratio of serum to cerebrospinal fluid (csf) *β*‐hCG can aid in the diagnosis of choriocarcinoma when imaging is inconclusive. Elevated serum *β*‐hCG levels and serum to csf *β*‐hCG ratio less than 60 in a patient with either a normal or abnormal pregnancy history may be indicative of metastatic choriocarcinoma.^16^

Treatment of choriocarcinoma with brain metastases includes chemotherapy and cranial irradiation. A craniotomy can be employed where indicated to provide acute decompression.^2^ Though highly malignant, it is very chemosensitive with about a 90% cure rate.^2^, ^4^ Combination chemotherapy regimens are used in the treatment of high‐risk GTN. The commonly used regimen is EMA‐CO (etoposide, methotrexate, actinomycin D, cyclophosphamide, and vincristine) with a long‐term survival rate as high as 95%.^2^ Methotrexate infusion at a dose of 1 g/m^2^ has been shown to facilitate penetration across the blood–brain barrier. In certain facilities, the administration of intrathecal methotrexate at a dosage of 12.5 mg has also been implemented.^2^

Other forms of regimen employed include MEA (methotrexate, etoposide, dactinomycin); EMA/EP (etoposide, methotrexate, dactinomycin/etoposide, cisplatin); and MEF (methotrexate, etoposide, 5‐FU).^17^

In some centers, the etoposide/cisplatin regimen is used as an induction regimen in patients with high tumor burden to avert the risk of sudden tumor collapse, which may precipitate severe complications such as myelosuppression, septicemia, and multiple organ failure. After the initial administration of etoposide/cisplatin, patients proceed to standard full‐dose chemotherapy with EMA‐CO once they are medically stable.

In our clinical setting, various factors such as medication costs, drug availability, and the adverse effects associated with the EMA‐CO regimen influence our management strategy. Consequently, patients are treated with etoposide and cisplatin at doses of 100 mg/m^2^, which have demonstrated efficacy in inducing remission. This therapeutic approach has exhibited efficacy in achieving remission within our patient cohort, aligning with documented success in literature.^18^ The EMA‐CO regimen is reserved for cases where normalization of β‐hCG levels is not achieved following treatment with etoposide and cisplatin.

The absence of serum hPL level documentation for case two upon her initial presentation, coupled with the lack of subsequent follow‐up on serum hPL levels, poses a limitation to the study.

In conclusion, the cases presented underscore the significance of considering choriocarcinoma in the differential diagnosis and conducting a thorough history and examination, especially in women of childbearing age presenting with unexplained intracerebral hemorrhage. Timely and intensive treatment can lead to improved prognosis and prevent fatal outcomes.

## AUTHOR CONTRIBUTIONS

**Seth Kyei‐Fram:** Data curation; formal analysis; investigation; writing – original draft; writing – review and editing. **Osei Yaw Asamoah:** Data curation; writing – original draft; writing – review and editing. **Martin Agyei:** Conceptualization; project administration; supervision; writing – review and editing. **Priscilla Abrafi Opare‐Addo:** Conceptualization; project administration; supervision; writing – review and editing.

## FUNDING INFORMATION

The authors received no financial support for the research, authorship, and/or publication of this article.

## CONFLICT OF INTEREST STATEMENT

The authors declared no potential conflicts of interest with respect to the research, authorship, and/or publication of this article.

## CONSENT

Written informed consent was obtained from the patient to publish this report in accordance with the journal's patient consent policy.

## Data Availability

The data that support the findings of this case series are available from the corresponding author upon reasonable request. Due to patient confidentiality and privacy concerns, some restrictions may apply to the availability of certain data points. Requests for access to the data should be directed to kyeiframseth@gmail.com.
